# The impact of physical activity on reading literacy in 9–10-year-old children: the mediating role of executive function

**DOI:** 10.1186/s12889-026-27525-8

**Published:** 2026-05-22

**Authors:** Qi Wang, Min Yang, Qin Sun, Chenle Wang, Pengfei Wen, Yulin Tian

**Affiliations:** 1 School of Physical Education, Yanshan University, Qinhuangdao, China; 2 Department of PE, Beijing Wuzi University, Beijing, China; 3 School of Physical Education, Guangzhou Sport University, Guangzhou, China

**Keywords:** Physical activity, 9–10 years old, Reading literacy, Executive function, Mediating effect

## Abstract

**Objective:**

This study aimed to investigate the influence of physical activity on reading literacy in children aged 9–10 years and to examine the mediating role of executive function.

**Methods:**

A cross-sectional study design was adopted, involving 293 children aged 9–10 years (Valid sample size: 282). Physical activity levels were assessed using the Physical Activity Questionnaire for Children (PAQ-C). Reading literacy (literary and informational texts) was evaluated using the PIRLS 2021 assessment. Core components of executive function—inhibitory control (Flanker task), working memory (1-back task), and cognitive flexibility (Trail Making Test, TMT)—were measured using experimental paradigms. The mediation effect was tested using the Bootstrap method with 5,000 resamples.

**Results:**

The gender distribution of the sample was balanced. For parental education, the highest proportion of parents held high school or vocational college degrees, and the majority of household incomes were concentrated in the middle-income bracket. The mean physical activity score for children was 2.97 ± 0.76, indicating a moderate-to-low level. Girls significantly outperformed boys in both literary (17.26 ± 4.84 vs. 16.27 ± 4.55) and informational (15.37 ± 4.63 vs. 14.13 ± 5.51) reading literacy. No significant gender differences were observed in any executive function subcomponents: inhibitory control (142.74 ± 81.53), working memory (1040.43 ± 86.10), or cognitive flexibility (93.99 ± 30.73). Additionally, parental education level and household income had significant effects on children’s physical activity, reading literacy, and executive function. Pearson correlation analysis revealed that physical activity was positively correlated with both literary (*r* = 0.341) and informational (*r* = 0.319) reading literacy, indicating weak positive correlations. Physical activity showed significant negatively correlated with all executive function subcomponents (inhibitory control: *r* =-0.457; working memory: *r* = -0.431; cognitive flexibility: *r* = -0.393), suggesting weak-to-moderate negative correlations. Both types of reading literacy were negatively correlated with all executive function subcomponents. (∣r∣= ranging from 0.293 to 0.457), also representing weak-to-moderate associations. The structural equation model indicated that the total effect of physical activity on reading literacy was 0.160, the direct effect was 0.093, and the indirect effect mediated by executive function was 0.070, accounting for 43% of the total effect.

**Conclusions:**

(1) Children’s physical activity level is positively correlated with reading literacy and can serve as a significant predictor of reading proficiency; (2) Executive function partially mediates the association between physical activity and reading literacy.

## Introduction

Regular participation in physical activity (PA) among school-aged children has been consistently shown to confer a wide range of health benefits across multiple domains, including improved cardiorespiratory fitness, regulation of metabolic homeostasis, and enhanced bone health [1–3]. However, global data from the World Health Organization (WHO) indicate that only 23.7% of children aged 11–17 meet the recommended daily level of 60 min of moderate-to-vigorous PA [4]. Recent surveys in China indicate that only approximately 32% of children aged 9–10 years meet the recommended daily level of physical activity [5]. Although governments and academic institutions increasingly emphasize the positive role of school-based physical activity—a subset of PA—in children’s physical and mental health, its promotion remains insufficient. Notably, given academic achievement is a fundamental objective of formal education, evidence indicating that PA positively predicts academic performance could substantially facilitate its implementation in both school and family environments. Therefore, investigating the facilitative effect of PA on academic achievement has become a focus of interdisciplinary research [6, 7].

Reading literacy—an integrated concept first introduced in 1991 by the International Association for the Evaluation of Educational Achievement (IEA) [8]—is defined as the ability to understand, use, and reflect on written texts to enable personal, social, and lifelong development. As a foundation of student core competencies, inadequate reading skills can significantly hinder educational and vocational progression [9, 10], and extensive experimental research confirms its critical role in learning efficacy and long-term development [11, 12].

Executive function (EF), is a higher-order cognitive capacity encompassing three core sub-dimensions—inhibitory control, working memory, and cognitive flexibility [13–16]. The age of 9–10 years represents a critical transition period for children from “learning to read” to “reading to learn,” as well as a sensitive stage for the development of executive function [17, 18]. Studies have shown that physical activity effectively promotes the development of executive function in children [19–21], while executive function subcomponents (such as inhibitory control, working memory, and cognitive flexibility) are deeply involved in the reading process [22–24]. Both EF and reading literacy are critical competencies for school-aged children, with sensitive developmental periods occurring during this same stage [25, 26], suggesting that the three may constitute a complex cascading mechanism. However, the existing literature has predominantly focused on pairwise relationships between variables (e.g., PA → EF, EF → reading literacy), leaving unclear whether PA enhances reading literacy through EF as a mediating pathway.

Accordingly, this study aims to systematically examine the mediating effect of EF in the relationship between PA and reading literacy in children by constructing a structural equation model, thereby revealing the underlying mechanisms among these three factors.

## Subjects and methods

### Subjects

The study focused on physical activity, executive function, and reading literacy in children aged 9–10 years. Using a cluster sampling method, all fourth-grade children (*n* = 293, age = 9.14 ± 0.32 years) from a public primary school in Qinhuangdao, Hebei Province, were selected as the participants. Based on school records and parental reports, this study excluded children with Attention Deficit Hyperactivity Disorder (A D H D), specific learning disorders, or other neurodevelopmental conditions that could significantly affect cognitive function. Additionally, children with uncorrected severe visual or hearing impairments, as well as those on long-term medication.

that might affect cognitive function or attention, were excluded. Prior to data collection, research permission was obtained from relevant school administrators and physical education teachers. Informed written consent was provided by all parents and legal guardians, and assent was obtained from all child participants.

Standardized data collection was conducted by trained researchers in December 2024. The process consisted of two phases: the first phrase involved administering physical activity questionnaires and collecting demographic information (Primarily including gender, parental education level, and household income); the second phase assessed reading literacy and executive function. Valid data were obtained from 282 children, comprising 149 boys and 133 girls.

### Research methods

#### Questionnaire survey

This study utilized the Physical Activity Questionnaire for Children (PAQ-C), a self-administered retrospective questionnaire developed by the University of Saskatchewan, Canada, and designed for children and adolescents. This instrument has been employed in physical activity level studies among Canadian children and adolescents, demonstrating good internal consistency and validity. The Chinese version, revised by Li et al., also exhibited acceptable reliability and validity (α = 0.85) and is suitable for large-sample physical activity research among Chinese youth [27].

The questionnaire consists of 10 items. The first nine items require children to self-report their physical activity over the past 7 days, aiming to measure habitual physical activity patterns rather than the intensity or duration of a single acute exercise bout or a long-term (e.g., months or years) history of sports training. Existing literature indicates that both long-term training and acute exercise have positive effects on executive function [28, 29] and reading literacy [30, 31]; therefore, the questionnaire’s lack of differentiation between acute and chronic activity has a minimal impact on the study’s core findings regarding the relationships among physical activity, executive function, and reading literacy. The tenth item assesses whether illness or other events prevented the child from participating in regular physical activity; however, this item is excluded from the calculation of the final activity score. For the composite physical activity score, each of the nine items (Items 1–9) is scored on a scale of 1 to 5. The final PAQ-C summary score is calculated as the mean of these nine items, with a score of 1 indicating a low level of physical activity and a score of 5 indicating a high level.

As the physical activity data in this study were collected via self-report, there was a potential risk of common method bias (CMB). To mitigate this bias, a dual control strategy was implemented: (1) During the survey administration, students were informed about the confidentiality of the data and assured that the information would be used solely for scientific research purposes; (2) Statistical methods were applied to test for common method bias. Harman’s single-factor test [32, 33], was primarily used, involving an unrotated principal component analysis of all variables in SPSS. The results indicated the extraction of three factors with eigenvalues greater than 1, with the largest factor accounting for 27% of the variance (below the critical threshold of 40%), suggesting the absence of severe common method bias in this study.

#### Testing methods

##### Reading literacy

The Progress in International Reading Literacy Study (PIRLS) is a globally influential research project on primary school students’ reading literacy, initiated and organized by the International Association for the Evaluation of Educational Achievement (IEA). Launched in 2001, PIRLS targets children aged 9–10, typically fourth-grade students, as this age represents a critical transition from learning to read to reading to learn. The assessment is conducted every five years, with the most recent cycle having taken place in 2021.

The test is divided into two sections, each requiring 40 min to complete, with a short break of approximately 5 to 15 min in between. The entire assessment takes approximately 90 min. It consists of two reading comprehension exercises: one based on informational text (e.g., expositions, instructions, data, and charts) and the other on literary text (e.g., stories, poems, and fables). The question types include multiple-choice questions, true/false items, short-answer questions, table completion, matching, and sequencing.

A dual scoring system is employed, in which two trained raters evaluate all responses. Prior to scoring, raters received standardized training to ensure consistent application of the scoring criteria. In cases of scoring discrepancies between the two raters, a third rater is engaged to resolve the disagreement, followed by a collaborative re-evaluation conducted by all three raters.

As an internationally recognized tool for assessing reading literacy, the PIRLS framework and items have been widely applied and validated in dozens of countries and regions, demonstrating high international comparability and good psychometric properties [34–37].

##### Executive function

Inhibitory control was assessed using the Flanker task, which measures conflict inhibition ability [38]. Working memory was evaluated using the 1-back task from the N-back paradigm, a measure of information maintenance and updating capacity. This paradigm was selected due to its appropriate cognitive load for children’s developmental level [39]. Cognitive flexibility was quantified using the Trail Making Test (TMT) to assess set-shifting efficiency [40].

The Flanker and 1-back tasks were administered via a computer using E-Prime software, whereas the TMT was completed in a paper-and-pencil format. Performance was assessed using reaction time (in milliseconds) as the primary metric, with shorter reaction times reflecting superior cognitive functioning.

To avoid handedness as a confounding variable in reaction time tasks, we screened all children using a brief handedness questionnaire, and all valid participants included in the final analysis were right-handed. Both reaction time and accuracy were recorded for all cognitive tasks; data from participants with an accuracy rate below 70% were excluded from the analysis [41] to ensure that the data reflected valid cognitive performance rather than random responding. Furthermore, to minimize the impact of extreme values, reaction times exceeding ± 3 standard deviations from the individual mean were removed as outliers prior to calculating the mean reaction time for each executive function component [42].

**Fig. 1:**
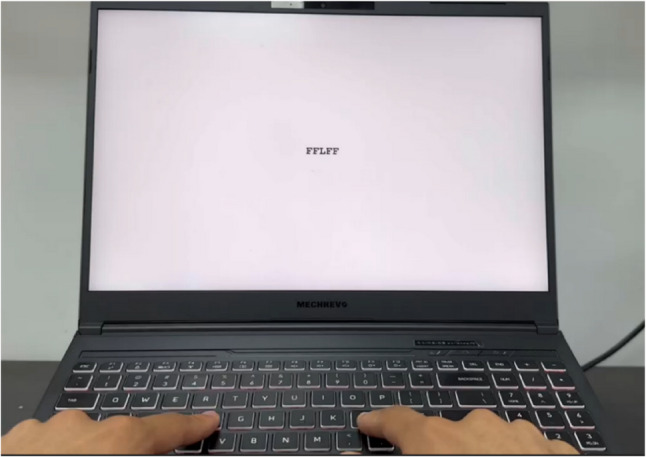
Diagram of the flanker task

- (1) Inhibitory control:

In the experimental task, a fixation point was presented for 0.5 s, followed by the display of a letter string on the screen for 1 s, with an inter-stimulus interval of 2 s. The task consisted of two types of trials: congruent trials (e.g., “FFFFF”, “LLLLL”) and incongruent trials (e.g., “LLFLL”, “FFLFF”). Participants were instructed to respond as quickly and accurately as possible to the central letter by pressing the “F” key with their index finger when the letter was “F”, or the “L” key when it was “L”. The experimenter guided participants through practice trials until they demonstrated a clear understanding of the procedure, after which the formal test commenced. The two conditions were presented with equal frequency and in randomized order. Reaction time and accuracy were recorded by the computer on each trial. (Fig. 1)

**Fig. 2:**
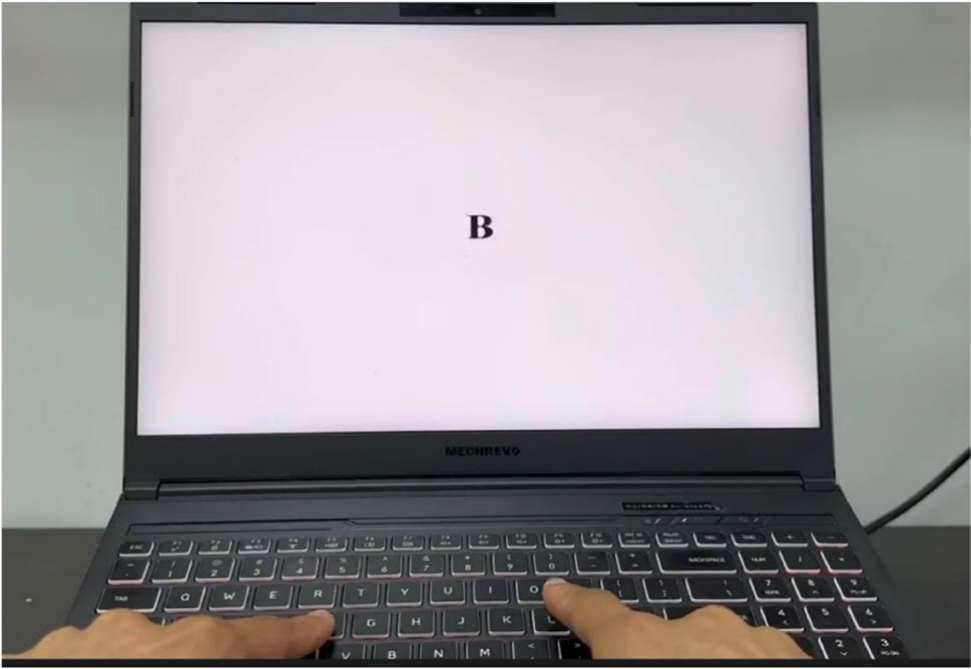
Diagram of the 1-back task

- (2) Working memory:

The 1-back task was administered using five English letters— B, D, L, Y, P—as stimuli. Each letter was presented individually at the center of the screen for 1 s, with an inter-stimulus interval of 3 s. Participants were instructed to carefully observe the letters and press the “L” key if the current letter matched the one presented immediately before it, or the “J” key if it did not. Reaction time and accuracy were recorded for each trial, and task performance was assessed based on the average reaction time. (Fig. 2)

**Fig. 3:**
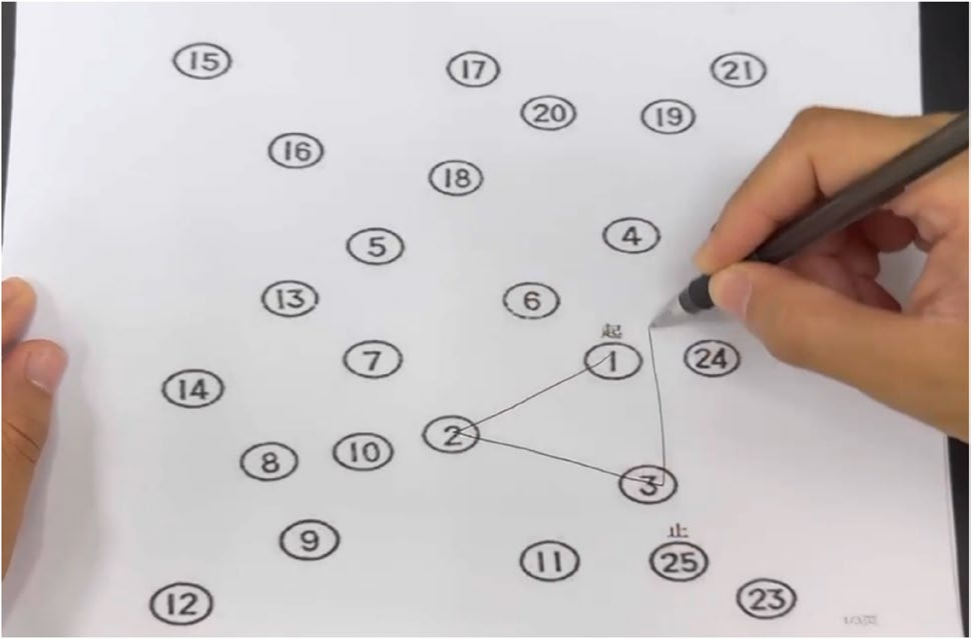
Diagram of the trail making test

- (3) Cognitive flexibility:

The test consists of two parts; Part A and Part B. In Part A, participants were required to connect 25 numbers in sequential order. Part B involves connecting a sequence numbers (1–13) and letters (A–L) in alternating order. (i.e., 1–A–2–B–3–C–……–12–L–13). The test score is calculated as the difference in mean reaction time between TMT-B and TMT-A. (Fig. 3)

#### Statistical analysis

The data collected after testing were analyzed using SPSS 26.0. The results are presented as mean ± standard deviation (M ± SD). First, continuous variables were tested for normality. Subsequently, independent samples t-tests were employed to compare differences in variables across genders, and one-way analysis of variance (ANOVA) was conducted to examine the effects of parental education level and household income on children’s physical activity levels, reading literacy, and executive function. On this basis, Pearson correlation analysis was performed to explore the relationships among physical activity, reading literacy, and executive function. Finally, structural equation modeling was constructed using AMOS 26 to test the mediating effect of executive function on the relationship between physical activity and reading literacy, with bootstrap resampling set to 5,000 repetitions and a 95% confidence interval (CI).

## Results and analysis

### Descriptive statistical analysis of variables

Skewness and kurtosis coefficients were used to test the normality of the data. Following Kline’s recommendation, data were considered not to deviate severely from a normal distribution if the absolute value of skewness was less than 2 and the absolute value of kurtosis was less than 7, making them suitable for subsequent parametric tests [43].

As shown in Table 1 the mean physical activity score was 2.97 (SD = 0.76), distributed across the full range of 1–5, indicating certain individual differences in physical activity levels within the sample. regarding reading literacy, the mean total score was 30.84 (SD = 9.13). Regarding the two sub-dimensions, children scored slightly higher on literary texts (M = 16.29, SD = 4.40) than on informational texts (M = 14.55, SD = 5.16). The three core components of executive function were measured using reaction time (lower values indicate better performance). Among them, inhibitory control had the longest mean reaction time and the greatest individual variability (M = 142.74 ms, SD = 81.53), followed by working memory (M = 1040.43 ms, SD = 86.10), while the mean completion time for cognitive flexibility was 93.99 s (SD = 30.73).

**Table 1:** Descriptive statistics of variables

Variable		Min	Max	Mean	SD	Skewness	Kurtosis
Physical Activity		1	5	2.97	0.76	-0.453	-0.802
Reading Literacy	Literary	6	22	16.29	4.40	0.651	0.432
	Informational	5	24	14.55	5.16	0.742	-0.413
Executive Function	Inhibitory Control (ms)	2.33	282.23	142.74	81.53	1.102	1.513
	Working Memory (ms)	900.23	1379.22	1040.43	86.10	0.658	0.603
	Cognitive Flexibility (s)	15	117	93.99	30.73	-0.807	0.903

### Analysis of demographic differences

#### Gender

As shown in Table 2, the sample contained slightly more boys than girls, displaying a generally balanced gender distribution. The tests for between-group differences in physical activity, inhibitory control, working memory, and cognitive flexibility all yielded *P* > 0.05 (0.567, 0.539, 0.900, and 0.534), indicating no statistically significant gender differences in these variables. In contrast, significant gender differences were observed for both literary reading literacy and informational reading literacy (*P* < 0.05; 0.042 and 0.048), suggesting that girls outperformed boys in reading literacy at the age of 9–10 years. Therefore, gender was included as a control variable in the subsequent data analyses.

**Table 2:** Analysis of gender differences in variables

Measure		Male(*n* = 149)	Female(*n* = 133)	*p*-value	t-value
Physical Activity		2.99 ± 0.77	2.94 ± 0.75	0.567	0.574
Reading Literacy	Literary	16.27 ± 4.55	17.26 ± 4.84	0.048*	-1.990
	Informational	14.13 ± 5.51	15.37 ± 4.63	0.042*	-2.043
Executive Function	Inhibitory Control (ms)	139.92 ± 82.70	145.91 ± 80.40	0.539	-0.615
	Working Memory (ms)	1039.23 ± 92.42	1040.57 ± 84.47	0.900	-0.126
	Cognitive Flexibility (s)	93.66 ± 31.89	95.95 ± 29.48	0.534	-0.623

Note: *p < 0.05

#### Parental education level

The results of the one-way ANOVA (see Table 3) indicated that the highest proportion of parents held high school or vocational college degrees, with a substantial proportion holding bachelor’s degrees or above, suggesting that the educational level of parents in the sample was generally above average. Parental education level had a significant impact on children’s physical activity, reading literacy (literary and informational), and executive function (inhibitory control, working memory, and cognitive flexibility). Post-hoc comparisons (LSD) revealed that children whose parents had a bachelor’s degree or higher significantly outperformed those in the lower education groups in physical activity levels, reading literacy (literary and informational), and executive function (inhibitory control, working memory, and cognitive flexibility). Therefore, parental education level should be included as a control variable in the model for subsequent data analyses.

**Table 3:** Analysis of differences by parental education level

Measure		Junior high & below (*n* = 74)	High school/Vocational(*n* = 122)	Bachelor’s & above (*n* = 86)	F (*p*)	Post-hoc (LSD)
Physical Activity		2.87 ± 0.81	2.95 ± 0.73	3.09 ± 0.74	4.895**	3>1
Reading Literacy	Literary	15.54 ± 4.61	16.78 ± 4.23	17.81 ± 3.63	5.950**	3>1, 3>2
	Informational	13.47 ± 5.60	14.57 ± 5.07	16.11 ± 4.48	5.504**	3>1, 3>2
Executive Function	Inhibitory Control (ms)	153.58 ± 84.31	144.11 ± 79.83	130.39 ± 80.71	5.655**	3<1, 3<2
	Working Memory (ms)	1058.08 ± 103.58	1039.14 ± 83.16	1023.60 ± 78.21	6.079**	3<1
	Cognitive Flexibility (s)	97.89 ± 29.64	96.36 ± 29.69	89.33 ± 32.93	4.857**	3<1

1 = Junior high school and below, 2 = High school or vocational college, 3 = Bachelor’s degree and above

*p < 0.05, **p < 0.01

#### Household income

As shown in Table 4 one-way ANOVA indicated that the majority of household incomes were concentrated in the middle-income bracket. Significant differences (*p* < 0.01) were observed among different household income groups in children’s physical activity, reading literacy (literary and informational), and executive function (inhibitory control, working memory, and cognitive flexibility). Post-hoc comparisons (LSD) revealed that the high-income group (100k and above) performed significantly better than the low-income group (0–30k) in physical activity, reading literacy, and executive function. Therefore, household income should be included as a control variable in the model for subsequent data analyses.

**Table 4:** Analysis of differences by household income

Measure		0–30k (*n* = 84)	30–100k (*n* = 145)	100k & above (*n* = 53)	F (*p*)	Post-hoc (LSD)
Physical Activity		2.85 ± 0.83	2.93 ± 0.69	3.27 ± 0.76	5.584**	3>1
Reading Literacy	Literary	14.89 ± 3.94	15.88 ± 4.46	16.13 ± 4.17	3.660**	3>1, 2>1
	Informational	14.78 ± 4.56	15.03 ± 5.52	16.75 ± 4.92	3.202**	3>1
Executive Function	Inhibitory Control (ms)	155.96 ± 90.22	146.32 ± 75.71	111.99 ± 76.00	5.161**	3<1, 2<1
	Working Memory (ms)	1052.88 ± 98.42	1043.06 ± 82.01	1010.48 ± 84.59	3.999**	3<1
	Cognitive Flexibility (s)	97.15 ± 33.03	92.35 ± 28.21	85.36 ± 32.40	3.124**	3<1

1 = 0–30k, 2 = 30k–100k, 3 = 100k and above

*p < 0.05, **p < 0.01

### Correlation analysis

As shown in Table 5, physical activity was significantly and weakly positively correlated with both literary reading literacy (*r* = 0.341, r^2^ = 0.116, *p* < 0.01) and informational reading literacy (*r* = 0.319, r^2^ = 0.102, *p* < 0.01). Physical activity was also significantly and weakly negatively correlated with all subcomponents of executive function—inhibitory control (*r* = -0.457, r^2^ = 0.209), working memory (*r* = -0.431, r^2^ = 0.186), and cognitive flexibility (*r* = -0.393, r^2^ = 0.154). Similarly, both literary and informational reading literacy were significantly and weakly negatively correlated with all executive function subcomponents (inhibitory control: *r* = -0.303, r^2^ = 0.092 and − 0.327, r^2^ = 0.107; working memory: *r* = -0.391, r^2^ = 0.153 and − 0.417, r^2^ = 0.174; cognitive flexibility: *r* = -0.293, r^2^ = 0.086 and − 0.325, r^2^ = 0.106, respectively). Based on these significant associations among the variables, this study constructed a mediation model with executive function as the mediator in the relationship between physical activity and reading literacy.

**Table 5:** Correlation matrix of variables

		Physical Activity	Reading Literacy		Executive Function		
			Literary	Informational	Inhibitory	Working	Cognitive
Physical Activity		1					
Reading Literacy	Literary	0.341**	1				
	Informational	0.319**	0.641**	1			
Executive Function	Inhibitory	-0.457**	-0.303**	-0.327**	1		
	Working	-0.431**	-0.391**	-0.417**	0.561**	1	
	Cognitive	-0.393**	-0.293**	-0.325**	0.431**	0.414**	1

r = correlation; * *p* < 0.05, ** *p* < 0.01

### Mediation effect test

**Fig. 4:**
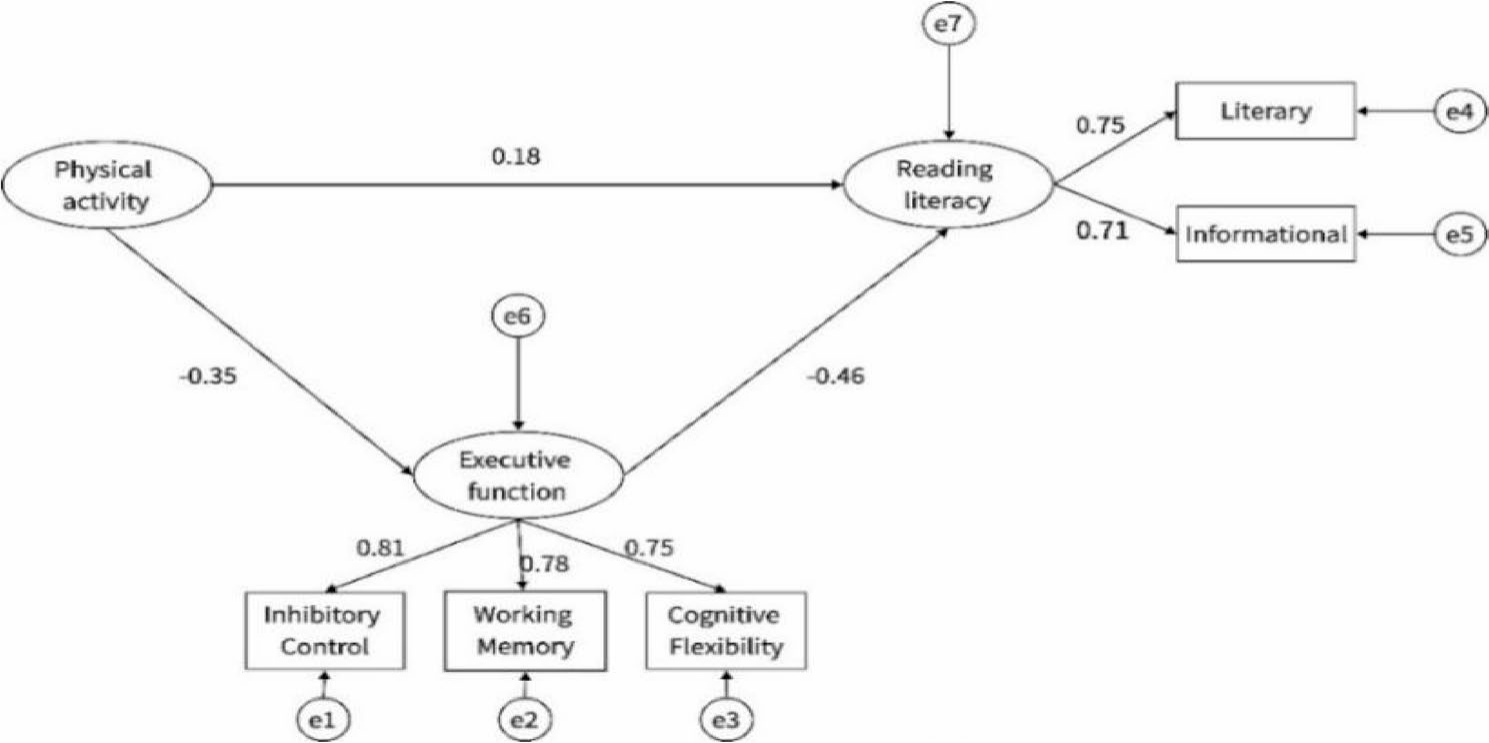
Mediation model of executive function between physical activity and reading literacy

Given the differences in children’s physical activity, reading literacy, and executive function across gender, parental education level, and household income, this study included these factors as control variables in the structural equation model. Initially, a structural equation model was constructed with physical activity level as the independent variable, reading literacy as the dependent variable, and executive function as the mediating variable(see Fig. 4). The model demonstrated a good fit and was deemed acceptable (CMIN/DF = 1.851, RMSEA = 0.045, CFI = 0.970, TLI = 0.965, IFI = 0.971), with all fit indices falling within the statistically recommended thresholds (see Table 6 for details).

**Table 6:** Model fit indices

Fit Index	Acceptable Criterion	Model Value
CMIN/DF	1–3 (Excellent), 3–5 (Good)	1.851
RMSEA	< 0.05 (Excellent), < 0.08 (Good)	0.045
CFI	> 0.90 (Excellent), > 0.80 (Good)	0.970
TLI	> 0.90 (Excellent), > 0.80 (Good)	0.965
IFI	> 0.90 (Excellent), > 0.80 (Good)	0.971

*CMIN/DF *Chi-square to Degrees of Freedom Ratio, *RMSEA *Root Mean Square Error of Approximation, *CFI *Comparative Fit Index, *TLI *Tucker-Lewis Index, *IFI *Incremental Fit Index

Results of the regression coefficients in the model indicated that physical activity level significantly and positively predicted reading literacy (PA → Reading Literacy = 0.181, *p* < 0.01); physical activity significantly and negatively predicted executive function (PA → Executive Function = -0.352, *p* < 0.01); and executive function significantly and negatively predicted reading literacy (Executive Function → Reading Literacy = -0.458, *p* < 0.01) (see Table 7).

**Table 7:** Path analysis results of the structural equation model

Path	Estimate	S.E.	C.*R*.	*P*
PA → Reading Literacy	0.181**	0.065	2.769	0.006
PA → Executive Function	-0.352**	0.055	-6.364	0.001
Executive Function → Reading Literacy	-0.458**	0.060	-7.502	0.001

*PA * Physical Activity, **P<0.01

The mediating effect was tested using the bootstrap method with 5,000 resamples. A significant effect was indicated if the 95% CI for the path coefficient did not include zero. The results showed that the total effect of physical activity on reading literacy was 0.163 (0.075, 0.245). The direct effect was 0.093, and the 95% CI for the direct effect point estimate (0.025, 0.135) did not include zero, indicating a significant direct effect. The indirect effect of physical activity on reading literacy through executive function was 0.070, and the 95% CI for the indirect effect point estimate (0.040, 0.120) did not include zero, indicating a significant indirect effect (see Table 8). These results demonstrate that physical activity not only directly enhances reading literacy but also influences it through the mediating role of executive function.

**Table 8:** Effect analysis

Path	Estimate	Lower	Upper	*P*	Proportion of Effect
Total Effect	0.163	0.075	0.245	0.001	
Direct Effect	0.093	0.025	0.135	0.005	57%
Indirect Effect	0.070	0.040	0.120	0.001	43%

## Discussion

### Current status of children’s physical activity, reading literacy, and executive function

The results of this study indicate that the total physical activity score of children was 2.97 ± 0.76, falling within a moderate-to-low range, with no significant gender differences observed. This suggests that gender does not significantly influence children’s current level of participation in physical activity, a finding consistent with studies by Zaccagni and Nouwen et al. [44–46]. It also implies that insufficient physical activity among Chinese children may be associated with public health issues such as declining physical fitness and rising obesity rates [47, 48]. Physical activity levels differed significantly by parental education level and household income. Extensive research indicates that family socioeconomic status (SES) is a key environmental factor influencing children’s physical activity participation [49, 50]. Higher parental education and household income often imply greater economic capital, health knowledge, and social resources, which can provide children with more opportunities to participate in organized sports and access exercise facilities [51, 52].

In terms of reading literacy, girls significantly outperformed boys in both literary texts (17.26 ± 4.84 vs. 16.27 ± 4.55) and informational texts (15.37 ± 4.63 vs. 14.13 ± 5.51), indicating a potential advantage for girls in narrative comprehension and expository text processing. This aligns with findings reported by David and Hanewinkel et al. [53, 54]. Girls’ superior reading performance may be related to their earlier language development [55, 56], suggesting the need for gender-adapted reading instruction strategies in the future. Reading literacy also showed significant differences based on parental education level and household income. High-SES families can not only provide richer home reading resources (e.g., books) and a more positive home literacy environment (e.g., parent-child reading), directly promoting the development of reading skills [57], but also exert an indirect influence on children’s reading habits and achievement through the “family cultural capital” formed by parents’ higher education levels, their values regarding education, and their educational involvement [58].

None of the executive function subcomponents-inhibitory control (142.74 ± 81.53), working memory (1040.43 ± 86.10), or cognitive flexibility (93.99 ± 30.73)—showed significant gender differences, which is consistent with conclusions from Bartgis and Jansen et al. [59, 60], This further indicates balanced development of executive function across both genders in the study population.

However, executive function performance was closely related to parental education level and family economic status. Children whose parents had a bachelor’s degree or higher performed better in inhibitory control, working memory, and cognitive flexibility. This may be because families with higher educational backgrounds and better economic conditions place greater emphasis on the systematic cultivation of children’s cognitive abilities. They are able to provide more diverse and high-quality cognitive stimulation (e.g., puzzle games, complex conversations, problem-solving opportunities) and a more stable emotional support environment, thereby more effectively promoting the development of executive function [61].

### Correlation analysis of children’s physical activity, reading literacy, and executive function

Pearson correlation analysis indicated that children’s physical activity was significantly positively correlated with both literary reading literacy (*r* = 0.341, *p* < 0.01) and informational reading literacy (*r* = 0.319, *p* < 0.01), which is consistent with the findings of Donnelly, Field, et al. [62–64], This supports the notion that higher levels of physical activity may simultaneously enhance both types of text processing abilities. Meanwhile, physical activity was significantly and negatively correlated with all subcomponents of executive function (inhibitory control: *r* = -0.457; working memory: *r* = − 0.431; cognitive flexibility: *r* = -0.393; all *p* < 0.01). This result aligns with studies by Hanewinkel, Campbell, et al. [46, 65], suggesting that physical activity optimizes cognitive resource allocation, thereby significantly reducing task completion time in executive function measures. Furthermore, reading literacy was significantly and negatively correlated with all executive function subcomponents (∣r∣ = 0.293–0.417), a finding consistent with research by Barber, Kieffer, et al. [66, 67], Children with higher executive function may enhance decoding automaticity, reduce cognitive load, and ultimately improve reading efficiency.

### The mediating effect of executive function between physical activity and reading literacy in children

Results from the structural equation modeling in this study indicate that physical activity not only directly predicts reading literacy but also indirectly influences it through executive function. Executive function played a partial mediating role between physical activity and reading literacy, accounting for 43% of the total effect. Possible underlying explanations: First, appropriate physical exercise can improve cardiorespiratory fitness, enhance cerebral blood flow, promote neurovascular coupling, and increase plasticity in the hippocampus and prefrontal cortex, thereby improving memory and learning efficiency [68, 69]. Second, physical activity enhances brain function through molecular mechanisms such as Brain-Derived Neurotrophic Factor (BDNF) and Insulin-like Growth Factor 1 (IGF-1), as well as structural improvements including synaptic plasticity and myelination [70, 71]. Third, physical activity boosts self-efficacy, reduces anxiety and depression, and indirectly optimizes learning motivation and classroom engagement [72, 73]. Fourth, physical activity reduces hyperactive and impulsive behaviors, extends attention span, and improves classroom task completion, thereby helping children maintain positive engagement and academic performance, which further enhances reading literacy [74, 75]. Given that this study utilized a cross-sectional design, the model results can only reveal associations between variables and cannot infer causal relationships.

The analysis in this section regarding the differential roles of EF sub-functions is primarily based on existing theoretical literature and the correlation patterns observed among the variables in this study; conclusions regarding causality require further verification through targeted experimental designs in future research. Regarding the differential roles of EF subfunctions in the relationship between physical activity and reading literacy, inhibitory control showed the most significant mediating effect between physical activity and literary reading. Its mechanism is mainly reflected in three aspects: First, physical activity enhances anterior cingulate cortex (ACC) function, improving children’s ability to suppress irrelevant environmental distractions (e.g., classroom noise) and internal interference (e.g., irrelevant thoughts) during reading, thereby allowing attentional resources to be more effectively focused on textual content [45]. Second, strong inhibitory control enables children to more quickly identify and suppress excessive attention to minor character details in literary texts, thereby concentrating limited cognitive resources on integrating key plot elements [76, 77]. Finally, the mediating effect of inhibitory control is moderated by gender and text type. Girls rely more on emotional resonance in narrative comprehension, with their inhibitory control primarily suppressing emotional interference; boys, in contrast, use it more to suppress external environmental distractions. This difference may stem from girls’ stronger bilateral temporal lobe activation during literary text processing [78]. Working memory exhibited a dual-pathway mediating role in the relationship between physical activity and reading literacy. On one hand, physical activity directly improves information maintenance capacity by increasing hippocampal volume and functional connectivity (e.g., a 22.3% improvement in N-back task performance) [79, 80]; on the other hand, expanded working memory capacity allows children to activate more semantic nodes (e.g., words, concepts, background knowledge) simultaneously during reading, facilitating the formation of coherent text representations [81]. Cognitive flexibility plays a core role in promoting the dynamic adaptation of reading strategies. When children switch from literary to informational texts (e.g., popular science articles), high cognitive flexibility enables them to quickly shift processing strategies—from emotion-oriented to logic-oriented processing. This ability is reflected in significantly shorter completion times on Part B-A of the TMT, indicating improved mental set-shifting efficiency [82].

Despite the important findings of this study, several limitations should be noted: (1) The cross-sectional design precludes confirming the causal pathway by which physical activity affects reading literacy through executive function; (2) The sample was drawn from a single public school in one city, lacking diversity in region, urban/rural settings, school types, and socioeconomic backgrounds, which limits the extrapolation of the conclusions to a broader population of children (especially those in rural areas or different educational systems); future research should employ multi-regional and multi-school sampling; (3) This study used the validated PAQ-C questionnaire to assess physical activity, which is a common and feasible tool for large-scale research on school-aged children [83, 84]; however, reliance on subjective self-reports remains a limitation. Future studies integrating objective devices such as accelerometers and heart rate monitors to quantify physical activity will provide a more robust and multi-dimensional revelation of the links between physical activity, physiological function, and reading ability; (4) The absence of neuroimaging techniques such as functional Near-Infrared Spectroscopy (fNIRS) limits the explanatory power regarding brain mechanisms; future research is recommended to include resting-state functional connectivity analysis. (5) This study excluded children with ADHD and other neurodevelopmental conditions. While this exclusion was necessary to avoid confounding effects on executive function and reading literacy, it also limits the generalizability of our findings to these populations. Given that executive function deficits are considered core or causal features of ADHD, future research should specifically examine how physical activity influences reading literacy in children with ADHD, potentially through subgroup analyses or targeted intervention studies.

## Conclusion

This study found that children’s physical activity level is positively correlated with their reading literacy (including both literary and informational texts) and serves as a direct predictor of reading proficiency. Furthermore, physical activity may indirectly promote reading literacy by enhancing executive function (inhibitory control, working memory, and cognitive flexibility). These findings reveal potential pathways of interaction among the variables, though the causal relationships remain to be verified in subsequent research.

## Data Availability

The datasets used and analysed during the current study are available from the corresponding author on reasonable request.
